# Aberrantly Over-Expressed TRPM8 Channels in Pancreatic Adenocarcinoma: Correlation with Tumor Size/Stage and Requirement for Cancer Cells Invasion

**DOI:** 10.3390/cells3020500

**Published:** 2014-05-23

**Authors:** Nelson S. Yee, Qin Li, Abid A. Kazi, Zhaohai Yang, Arthur Berg, Rosemary K. Yee

**Affiliations:** 1 Division of Hematology-Oncology, Department of Medicine, Penn State College of Medicine, Program of Experimental Therapeutics, Penn State Hershey Cancer Institute, Penn State Milton S. Hershey Medical Center, Pennsylvania State University, Hershey, PA 17033, USA; E-Mails: QLi4@mdanderson.org (Q.L.); aak3@psu.edu (A.A.K.); 2 Division of Anatomic Pathology, Department of Pathology, Penn State College of Medicine, Penn State Milton S. Hershey Medical Center, Pennsylvania State University, Hershey, PA 17033, USA; E-Mail: zyang2@hmc.psu.edu; 3 Division of Biostatistics, Department of Public Health, Penn State College of Medicine, Penn State Milton S. Hershey Medical Center, Pennsylvania State University, Hershey, PA 17033, USA; E-Mail: aberg1@hmc.psu.edu; 4 Schreyer Honors College, Pennsylvania State University, University Park, PA 16802, USA; Penn State Harrisburg School of Humanities, Pennsylvania State University, Middletown, PA 17057, USA; E-Mail: rky5018@psu.edu

**Keywords:** biomarker, cell invasion, cell migration, ion channel, molecular target, pancreatic cancer, TRPM8, transient receptor potential

## Abstract

The transient receptor potential melastatin-subfamily member 8 (TRPM8) channels control Ca^2+^ homeostasis. Recent studies indicate that TRPM8 channels are aberrantly expressed and required for cellular proliferation in pancreatic adenocarcinoma. However, the functional significance of TRPM8 in pancreatic tissues is mostly unknown. The objectives of this study are to examine the expression of TRPM8 in various histopathological types of pancreatic tissues, determine its clinical significance in pancreatic adenocarcinoma, and investigate its functional role in cancer cells invasion. We present evidence that, in normal pancreatic tissues, anti-TRPM8 immunoreactivity is detected in the centroacinar cells and the islet endocrine cells. In pre-malignant pancreatic tissues and malignant neoplasms, TRPM8 is aberrantly expressed to variable extents. In the majority of pancreatic adenocarcinoma, TRPM8 is expressed at moderate or high levels, and anti-TRPM8 immunoreactivity positively correlates with the primary tumor size and stage. In the pancreatic adenocarcinoma cell lines that express relatively high levels of *TRPM8*, short hairpin RNA-mediated interference of *TRPM8* expression impaired their ability of invasion. These data suggest that aberrantly expressed TRPM8 channels play contributory roles in pancreatic tumor growth and metastasis, and support exploration of TRPM8 as a biomarker and target of pancreatic adenocarcinoma.

## 1. Introduction

The goal of this study is to elucidate the clinical and functional significance of the transient receptor potential TRPM8 ion channel in pancreatic cancer. Pancreatic adenocarcinoma, the most common histopathological type of cancer in the pancreas, is highly lethal, and the prognosis of patients with pancreatic cancer is generally poor [1]. For the majority of patients with pancreatic adenocarcinoma, the disease is often diagnosed at the advanced stages, when palliative systemic chemotherapy may provide limited benefits. Development of biomarkers-based targeted agents has been under active investigation, and such therapeutics may offer new opportunities to improve treatment response in patients with pancreatic cancer [2]. Accumulating evidence has revealed the important roles of ion channels in cancer including those of the transient receptor potential (TRP) family [3,4]. Certain members of the TRP melastatin (TRPM) subfamily of ion channels are implicated in various human malignancies, and their expression and roles in pancreatic cancer have recently been identified [5,6]. Understanding the functions of these channels and their underlying mechanisms is expected to shed new light into the pathogenesis of pancreatic neoplasms and facilitate development of clinical biomarkers and therapeutic targets.

Translation of developmental regulators of exocrine pancreatic development in zebrafish has led to discovery of TRPM7 and its subfamily member TRPM8 in pancreatic adenocarcinoma [5,7,8,9,10,11]. TRPM8 channels are non-selective, voltage-gated, and permeable to divalent and monovalent ions [12,13]. TRPM8 channels can be activated by cold temperature or cooling compounds, leading to an increase in intracellular concentration of Ca^2+^ [9]. In human adult tissues, TRPM8 is selectively expressed and aberrantly expressed in carcinoma arising in various organs [6,14].

In human pancreatic adenocarcinoma cell lines and in a few specimens of pancreatic adenocarcinoma tissues examined, expression of TRPM8 is consistently elevated [7,9]. TRPM8 channels are required for maintaining proliferation and preventing replicative senescence of pancreatic cancer cells [7,9]. However, expression of TRPM8 channels in various pancreatic neoplasms, their clinical significance, and functional roles in pancreatic cancer are mostly unknown. Whether TRPM8 channels play a role in pancreatic cancer cells invasion has not been studied.

In this study, we aimed to examine the expression of TRPM8 in normal and pathological pancreatic tissues, explore the clinical significance of TRPM8 in pancreatic adenocarcinoma, and determine its role in cancer cells invasion. We analyzed the expression of the TRPM8 channels using microarrays of adult human pancreatic tissues by immunohistochemistry (IHC). Our data indicate TRPM8 channels are aberrantly over-expressed in the majority of specimens of pancreatic adenocarcinoma, and the expression levels of TRPM8 positively correlate with tumor size and stage. In pancreatic adenocarcinoma cell lines, short hairpin RNA-mediated silencing of *TRPM8* impaired cancer cells invasion. Results of this study suggest that aberrantly expressed TRPM8 channels play contributory roles in pancreatic tumor growth and metastasis. These data further support the potential of TRPM8 for development as a clinical biomarker and a therapeutic target in pancreatic adenocarcinoma.

## 2. Experimental Section

### 2.1. Pancreatic Tissue Microarrays

The human pancreatic tissues microarrays were obtained from Biomax, Inc. (Rockville, MD, USA). These microarrays contain normal and pathological pancreatic tissues, as well as a variety of pancreatic tumors. Demographic data such as age and gender are available. The pathological features including histopathology, tumor grade, tumor size, tumor extent, involvement of regional lymph nodes, and distant metastasis are described. Pathological staging of pancreatic adenocarcinoma is based on the American Joint Cancer Commission 7^th^ edition. The therapeutic modalities, treatment response, and survival data that correspond to the tissue microarrays are not provided by Biomax, Inc.

### 2.2. Histological and Immunohistochemical Analysis of Anti-TRPM8 Immunoreactivity

The tissue microarray slides were processed for staining with hematoxylin and eosin using standard procedures. Expression of TRPM8 was analyzed by IHC using rabbit polyclonal anti-human TRPM8 antibodies (Lifespan Biosciences, Inc., Seattle, WA, USA) at a 1:100 dilution, followed by incubation with horseradish peroxidase-conjugated anti-rabbit IgG (EnVision™ + System, Dako, Carpinteria, CA, USA). The signals were detected by color reaction using 3,3’-diaminobenzidine (Dako), counterstained with hematoxylin (Richard-Allan Scientific^®^/VWR, Radnor, PA, USA), and mounted using Permount (Sigma-Aldrich^®^, St. Louis, MO, USA). The brown color indicates immunoreactivity against TRPM8 protein. Tissue sections incubated in the absence of anti-TRPM8 antibodies were processed in parallel, and they did not exhibit any detectable TRPM8-specific immunoreactivity (data not shown).

Each tissue section is present in triplicates. Each specimen was examined under a compound light microscope to determine the histopathological features and the level of expression of TRPM8. The pathologist (Z.Y.) and the medical oncologist (N.S.Y.) were blinded from the clinicopathological data at the time of immunohistochemical examination. The level of expression of TRPM8 is based on immunoreactivity using a conventionally used scoring system of 0 to 4+ and the percentage of positive cells. (0 to 1+ and <50%) is considered as no-to-low expression; (1+ and ≥50% to 2+ and 100%), moderate expression; (≥3+ and any %), high expression. Images were acquired under a compound light microscope using a digital camera (DP12, Olympus, Center Valley, PA, USA) and analyzed with Adobe^®^ Photoshop^®^ Elements.

### 2.3. Statistical Analysis

The expression level of TRPM8 was determined by IHC, which was scored by multiplying the intensity (range 0–4) with the percentage of positive cells (range 0%–100%). The distribution of the IHC score against age, gender, primary tumor size, histological grade, and stage are displayed along with the respective means, standard errors, and *p*-values. The statistical significance was assessed using linear regression treating the IHC score as the response variable. The ordered categorical variables (primary tumor size, histological grade, and stage) were analyzed with an interval scale (ranging from 1 to 4 for each variable). Therefore, *p*-values for the ordered categorical variables are analogous to a trend test and the *p*-values for the binary variables (age >55, gender) are analogous to a two-sample *t*-test. The statistical analysis was performed using R [15]. The graphics were produced using the beeswarm package [16] in R.

Six pairwise comparisons were performed on the log-transformed IHC score data using the pairwise-*t*-test function in R with Bonferonni adjustments to the *p*-values to account for multiple comparisons. Polyserial correlation between the ordinal variables of primary tumor size/stage and the log-transformed IHC score data was calculated using the polyserial function in the polycor library in R. Statistical significance is determined with a two-sided permutation test using 10^5^ permutations.

### 2.4. Cell Lines and Culture

The human pancreatic adenocarcinoma cell lines (BxPC-3 and MIA PaCa-2) were obtained from American Type Culture Collection (ATCC, Manassas, VA, USA) and maintained according to ATCC’s instructions. The cells were incubated at 37 °C in a humidified atmosphere containing 5% CO_2_. They were used within twenty passages and periodically recovered from the stocks frozen in liquid nitrogen.

### 2.5. Immunoblot Analysis of TRPM8 Protein

For each cell line, 2.5 × 10^6^ cells were seeded in a 10-cm cell culture dish (Corning Inc., Corning, NY, USA) and incubated for 48 h. Cells were lyzed in RIPA buffer (Sigma-Aldrich^®^) containing EDTA-free mini-protease inhibitor cocktail (Roche Diagnostics, Indianapolis, IN, USA). The lysates were sonicated for 10 min, mixed for 30 min at 4 °C, and then centrifuged at 14,000× g for 10 min at 4 °C.

For each protein sample, equal volume was loaded in each lane in duplicates and analyzed using 10% SDS-PAGE. The gel was run overnight, and the proteins were transferred to polyvinylidene fluoride membrane (Biotrace; PALL, Pensacola, FL, USA). The membrane was blocked in 5% non-fat dry milk and incubated overnight at 4 °C with anti-human TRPM8 (rabbit polyclonal) or anti-human GAPDH (mouse monoclonal) antibodies (OriGene Technologies, Rockville, MD, USA) used at 1:750 and 1:2500 dilution, respectively. Excess primary antibodies were removed by washing the membrane in Tris-buffered saline containing 0.1% Tween 20. The membranes were then incubated with horseradish peroxidase–conjugated goat anti-rabbit or goat anti-mouse secondary antibodies (Bethyl Laboratories, Montgomery, TX, USA) at room temperature for 1 h. The membranes were rinsed with Tris-buffered saline containing 0.1% Tween 20 to remove excess secondary antibodies. TRPM8 and GAPDH proteins were detected by using enhanced chemiluminescence (ECL plus, Amersham, Piscataway, NJ, USA) according to the manufacturer’s instructions. The chemiluminescent signals were developed using a ProteinSimple Fluorchem M imaging system (Santa Clara, CA, USA). GAPDH was assayed as internal control for equal loading of protein samples. The protein levels of TRPM8 were normalized with those of GAPDH by quantifying the protein levels in the uncompressed images using the National Institutes of Health (NIH) ImageJ 1.6 software.

### 2.6. Short Hairpin RNA (shRNA)-Mediated Silencing of TRPM8

Four plasmids containing different shRNA directed against human *TRPM8* and a plasmid containing non-targeting shRNA were obtained from Superarray Biosciences/Qiagen (Valencia, CA, USA) and tested for efficiency of gene silencing. An shRNA under control of U1 promoter and green fluorescent protein (GFP) reporter gene are contained in each plasmid. The sequences of the anti-*TRPM8* shRNA were:

5'-AAACTTAGGACCCAAGATTAT-3'; 5'-AAGGAACTCTCCAAAGTCATT-3';
5'-AAACACCCAACCTGGTCATTT-3'; 5'-CAACGACACCTCAGAGGAAAT-3'.

Non-targeting control shRNA: 5'-ggaatctcattcgatgcatac-3'.

BxPC-3 cells at 90% confluency in a 10-cm dish containing 10 mL OptiMem^®^ medium (Invitrogen™, Life Technologies) were transfected with either 24 µg anti-*TRPM8* shRNA (Qiagen) or non-targeting control shRNA (Qiagen) using 60 µL Lipofectamine™ 2000 (Invitrogen™, Life Technologies, Grand Island, NY, USA) and then incubated at 37 °C for 5 h. The transfected cells were incubated in RPMI 1640 medium containing 10% fetal bovine serum (FBS, Life Technologies) at 37 °C for 24 h, sorted and collected by flow cytometry for green fluorescent protein (GFP) with emission light at a wavelength of 488 nm using BD FACSCalibur^®^ (BD Biosciences, San Jose, CA, USA). The transfected cells were incubated in culture medium at 37 °C for 24 h. Total RNA was extracted from the GFP-sorted cells and analyzed for *TRPM8* mRNA by reverse transcription followed by semi-quantitative polymerase chain reaction (PCR) as described [8]. Among the four anti-*TRPM8* shRNA, the shRNA targeting the sequence 5'-CAACGACACCTCAGAGGAAAT-3' produced the most repression of *TRPM8* mRNA (70% reduction) and it was used for further experiment.

### 2.7. *In Vitro* Assay for Cell Invasion

BxPC-3 and MIA PaCa-2 cells transfected with either anti-*TRPM8* shRNA (Qiagen) or non-targeting control shRNA (Qiagen) were incubated in RPMI 1640 medium containing 10% FBS at 37 °C for 24 h. The cells were washed in phosphate-buffered saline, and incubated in serum-free medium that contained 0.1% bovine serum albumin (Sigma^®^) for another 48 h. The cells were then seeded at 3 × 10^4^ cells in 1 mL medium in each well of a 6-well culture plate (Corning) with 10% Matrigel™ (BD Biosciences, San Jose, CA, USA)-coated trans-well inserts with 8 µm filters (Greiner Bio-One, VWR, Radnor, PA, USA), and 2.5 mL of 10% FBS (as a chemoattractant) in the lower chamber. Following incubation at 37 °C for 24 h, the cells were analyzed for invasion.

The cells that had migrated and invaded onto the lower side of the insert membrane were fixed with pre-cooled (4 °C) methanol and stained with 0.1% solution of crystal violet (Sigma^®^). The images were acquired under an inverted light microscope with phase contrast (Nikon ECLIPSE Ti, Melville, NY, USA) and processed using Adobe^®^ Photoshop^®^ 7. For each well, the cells that had invaded in each of 9 visual fields observed at 40× and 200× magnification were counted. A graph that shows invasion of the *TRPM8*-silenced cells through the trans-well was expressed as percentage of cells transfected with non-targeting control shRNA. Each experiment was conducted for a total of three times with similar results. Cell viability was determined by exclusion of trypan blue.

## 3. Results and Discussion

### 3.1. Expression of TRPM8 in Normal and Non-Malignant Pancreatic Tissues

TRPM8 channels are cellular sensors and regulators of Ca^2+^ homeostasis, and they have been implicated in human malignancies. We have recently demonstrated increased expression of TRPM8 in pancreatic adenocarcinoma cell lines and a few tissue specimens. However, the clinicopathological features and significance of TRPM8 in pancreatic adenocarcinoma and non-malignant pancreatic tissues as well as the various histologic subtypes of pancreatic tumors remain to be explored. Tissue microarrays from human pancreas were examined for expression of TRPM8 by IHC using specific anti-TRPM8 antibodies. There are 366 specimens, including normal pancreatic tissues, chronic pancreatitis, pre-malignant neoplasms, and a variety of malignant tumors. The various histopathological types of pancreatic tumors in these tissue microarrays are present in proportions that are representative of clinical situations.

Pancreatic adenocarcinoma, the most common type of cancer in the pancreas, is present in the largest proportion, whereas the other relatively rare malignant tumors are present in variably small proportions. There is relatively small number of metastatic pancreatic adenocarcinoma in the tissue microarrays as compared to that of primary pancreatic adenocarcinoma. This is because most of the primary pancreatic adenocarcinoma are surgically resected specimens, whereas the metastatic tumors are typically aspirated using a fine needle and not amenable to IHC. However, validity of the expression analysis is supported by the fact that, each patient’s pancreatic tissue is displayed as triplicates in the microarrays, and the immunohistochemical analysis of TRPM8 in the triplicate samples of each specimen is consistent throughout this tissue microarray.

**Figure 1:**
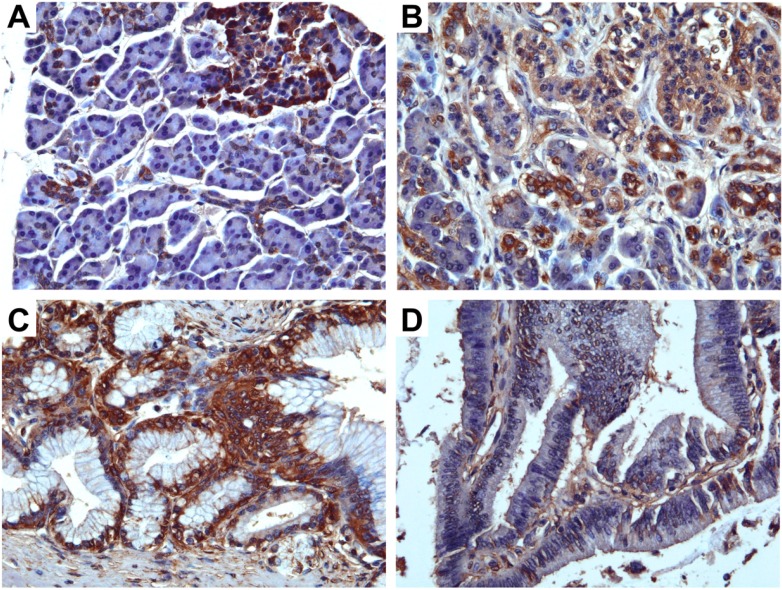
Immunohistochemistry using anti-TRPM8 in pancreatic tissues. (**A**) Normal pancreatic tissues. (**B**) Chronic pancreatitis. (**C**) Pancreatic intra-epithelial neoplasm. (**D**) Intraductal papillary mucinous neoplasm.

First, we examined the expression of TRPM8 in normal pancreatic tissues, chronic pancreatitis, and the pre-malignant lesions including pancreatic intra-epithelial neoplasms (PanINs) and intraductal papillary mucinous neoplasms (IPMN). In normal pancreatic tissues, TRPM8 is expressed in the ductal cells and centroacinar cells (Figure 1A). There is strong immunoreactivity against TRPM8 in the islet endocrine cells in which the expression of TRPM8 appears eccentric (Figure 1A). No appreciable anti-TRPM8 immunoreactivity was detectable in the acinar cells (Figure 1A).

In chronic pancreatitis, not only does anti-TRPM8 immunoreactivity becomes prominent in the ductal cells and centroacinar cells, however, it is also detectable in the acinar cells (Figure 1B). In PanINs, low-to-moderate levels of expression of TRPM8 are present in the basal plasma membrane of the ductal cells (Figure 1C). In intraductal papillary mucinous neoplasms (IPMN), a low level of anti-TRPM8 immunoreactivity is detected at the apical plasma membrane of the ductal cells (Figure 1D).

Thus, expression of TRPM8 protein in normal human pancreatic tissues is cell-specific, and it is aberrantly up-regulated in chronic pancreatitis and PanINs. It is unclear what the normal functions of the TRPM8 channels in the pancreatic epithelia. We speculate that the TRPM8 channels’ ability of sensing alterations in temperature and acidity contributes to the regulation of the physiological functions of exocrine and endocrine pancreas. Besides, whether TRPM8 plays a role in the formation of those pathological conditions of exocrine pancreas remains to be determined.

### 3.2. Expression of TRPM8 in Malignant Pancreatic Neoplasms

**Figure 2:**
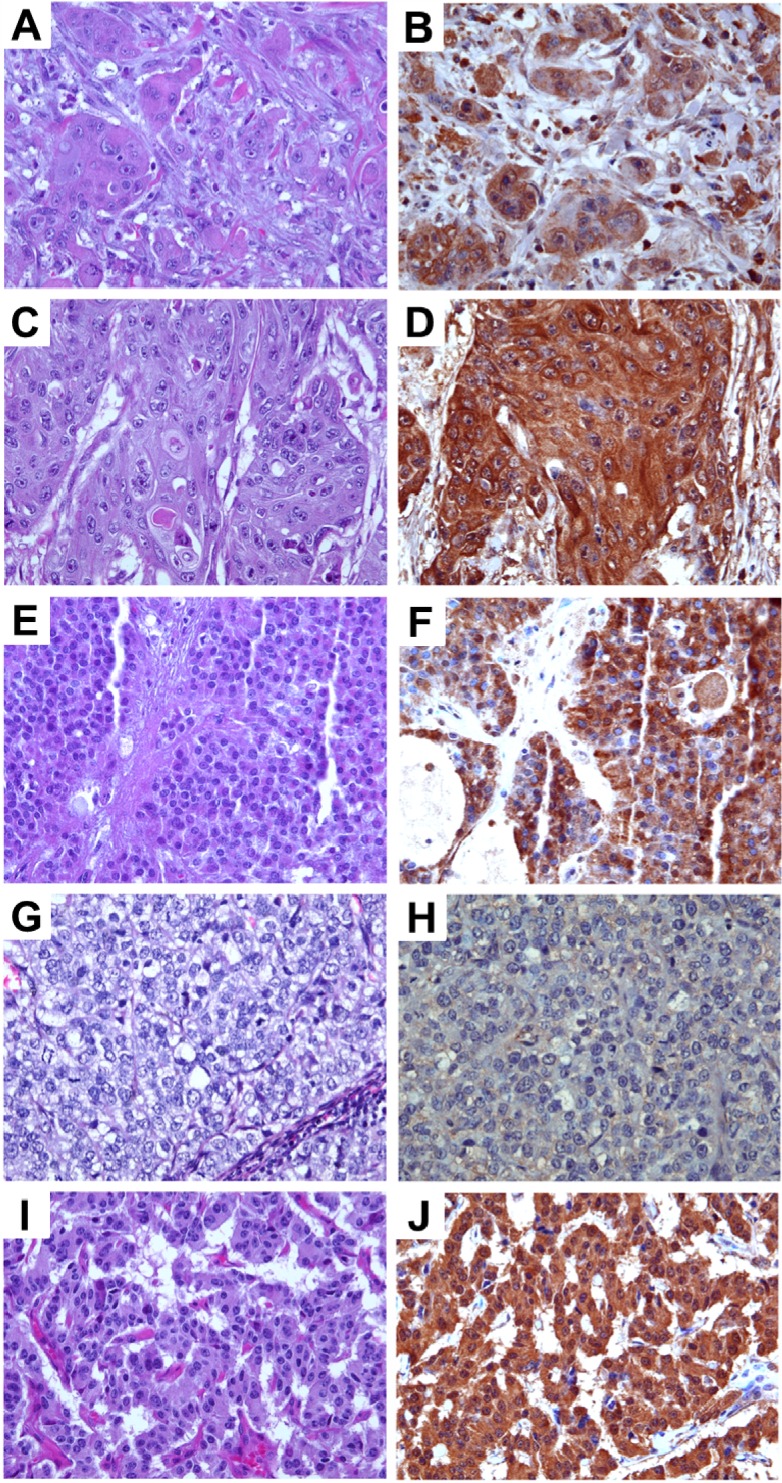
Immunohistochemical analysis of TRPM8 in malignant pancreatic tumors. (**A**,**B**) Adenocarcinoma. (**C**,**D**) Adenosquamous carcinoma. (**E**,**F**) Solid pseudo-papillary neoplasm (**G**,**H**) Acinar cell carcinoma. (**I**,**J**) Neuroendocrine tumor. (**A**,**C**,**E**,**G**,**I**) H and E, original magnification ×200. (**B**,**D**,**F**,**H**,**J**) Immunohistochemistry using anti-TRPM8 antibodies, original magnification ×400.

Various histopathological subtypes of malignant pancreatic tumors including adenocarcinoma, adenosquamous carcinoma, solid pseudopapillary carcinoma, and acinar carcinoma were examined for anti-TRPM8 immunoreactivity. There is moderate or high level of expression of TRPM8 in 92% (*n* = 258) of pancreatic adenocarcinoma (Figure 2A,B). Anti-TRPM8 immunoreactivity in the adenocarcinoma cells appears to be diffuse within the cells (Figure 2B).

In both adenosquamous carcinoma and solid pseudopapillary carcinoma, there are moderate-high levels of anti-TRPM8 immunoreactivity, which appears diffuse in the cytoplasm with accentuation in the plasma membrane (Figure 2C–F). No appreciable anti-TRPM8 immunoreactivity can be detected in all of the specimens of acinar carcinoma being examined (Figure 2G,H). In pancreatic neuroendocrine tumors, the majority of specimens express moderate anti-TRPM8 immunoreactivity (Figure 2I,J). It is noteworthy that, in pancreatic neuroendocrine tumor cells, expression of TRPM8 is diffuse within the cytoplasm (Figure 2J). This is in contrast to the eccentric distribution of TRPM8 protein in normal endocrine cells of pancreatic islets (Figure 1A). The proportions of each histological type of pancreatic neoplasms and the corresponding expression levels of TRPM8 are listed in Table 1. Besides adenocarcinoma, the other types of pancreatic tumors are relatively rare and present in small numbers. Further analysis that involves a large number of specimens will be necessary to determine the clinical significance of TRPM8 in those relatively rare pancreatic tumors.

**Table 1:** Expression of TRPM8 in various types of histopathology in pancreatic tumors with regard to the intensity of immunoreactivity and the percentage of positive cells. The total number of cases examined is 308. The values represent the number of specimens with the corresponding proportions in parentheses.

Pancreatictumors	Expression levels of TRPM8		
	No-to-low (0 to 1+, <50%)	Moderate (1+, ≥50% to 2+, 100%)	High (≥3+, any %)
Adenocarcinoma	22 (7.9%)	196 (70.0%)	62 (22.1%)
Adenosquamous carcinoma	0 (0.0%)	4 (57.1%)	3 (42.9%)
Solid pseudopapillary neoplasm	0 (0.0%)	1 (50.0%)	1 (50.0%)
Acinar cell carcinoma	3 (100.0%)	0 (0.0%)	0 (0.0%)
Neuroendocrine tumor	6 (37.5%)	10 (62.5%)	0 (0.0%)

### 3.3. Expression of TRPM8 Correlates with Clinicopathological Features of Pancreatic Adenocarcinoma

To understand the significance of TRPM8 in pancreatic adenocarcinoma, we analyzed the expression of TRPM8 in further detail and correlated it with the clinical and pathological features. There are 280 specimens of pancreatic adenocarcinoma in the tissue microarrays. The range of age is 23 to 78 years-old, mean age 56.5 ± 10.4. The corresponding clinical data are described in Table 2.

**Figure 3:**
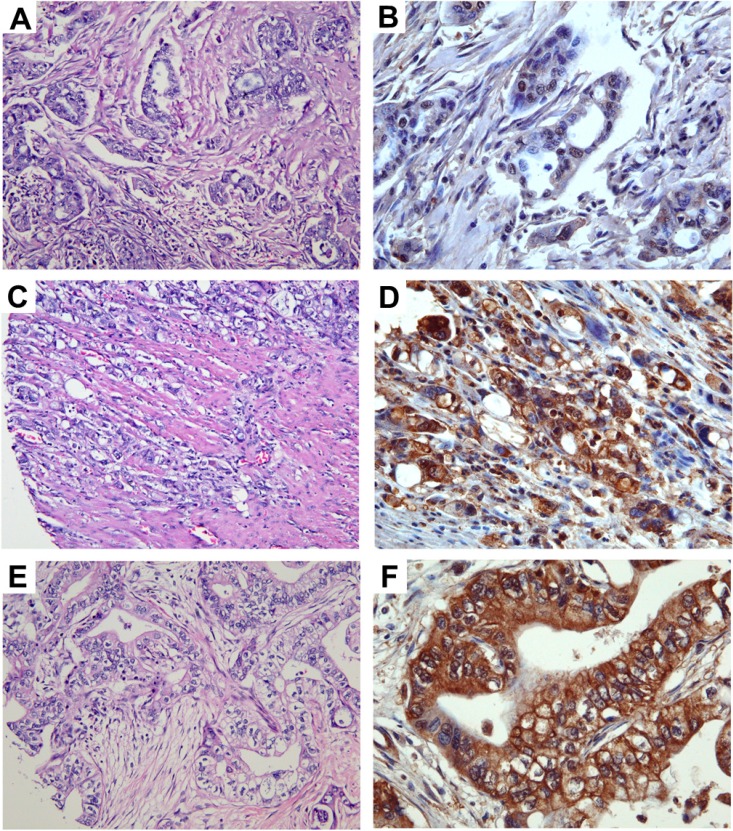
Expression levels of TRPM8 in pancreatic adenocarcinoma. Images in (**A**,**C**,**E**) (original magnification ×200) represent the H&E sections of the same tumor as the TRPM8 immunohistochemical staining in (**B**,**D**,**F**) (original magnification ×400), respectively. Expression levels of TRPM8: (**B**) no-to-low; (**D**) moderate; (**F**) high.

**Figure 4:**
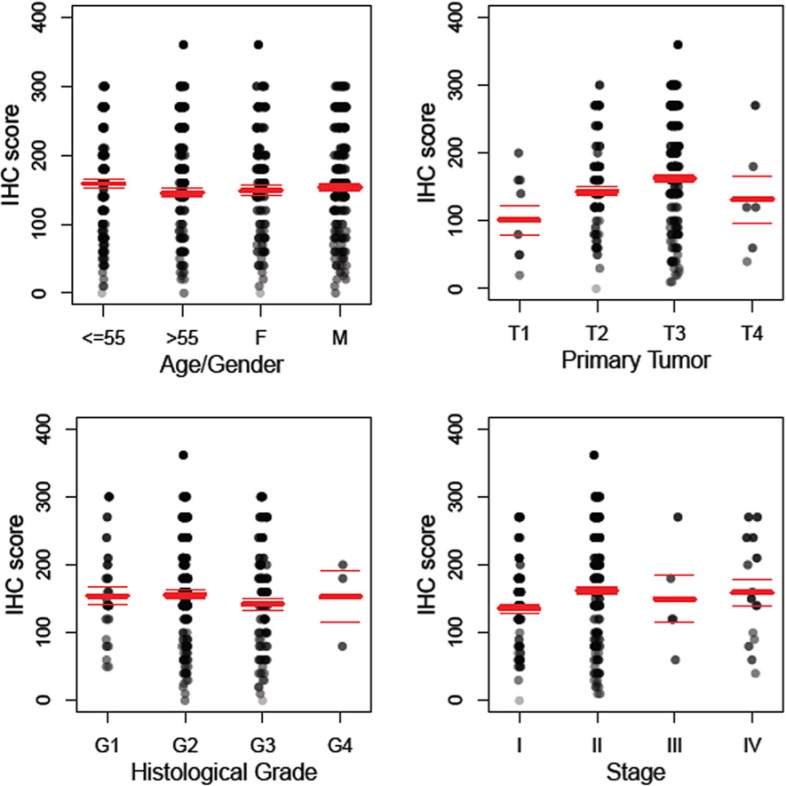
Anti-TRPM8 immunoreactivity in pancreatic adenocarcinoma positively correlates with primary tumor size and stages. The distribution of the IHC score for expression of TRPM8 is displayed against age/gender, histological grade, primary tumor (T), and stage. The total number of specimens of pancreatic adenocarcinoma examined is 280. Polyserial correlation between the primary tumor size (T) or the tumor stages and the log-transformed IHC score is 0.13 and 0.10, respectively.

More than 90% of the 280 specimens of pancreatic adenocarcinoma exhibit either moderate or high level of anti-TRPM8 immunoreactivity (Figure 3). The expression levels of TRPM8 positively correlate with the size of the primary tumor, as well as with the tumor stages (Figure 4). By pairwise comparisons using *t*-tests with pooled standard deviation, anti-TRPM8 immunoreactivity tends to be higher in the primary tumor at T3 than that at T1 (*p* = 0.08). Using this method of analysis, the expression levels of TRPM8 in tumors of stage II are higher than that of stage I, but the difference is not statistically significant (*p* = 0.39). There is no significant correlation between the expression levels of TRPM8 and the patients’ age, gender or the histological grades. Expression of TRPM8 is not significantly different in metastatic pancreatic adenocarcinoma as compared with that in the primary tumor.

The positive correlation of the expression levels of TRPM8 in pancreatic adenocarcinoma with tumor size and stages suggests that aberrant over-expression of TRPM8 is associated with tumor growth and metastasis. Up-regulated expression of TRPM8 has also been shown in other cancer tissues including that of prostate, breast, lung, colon/rectum, and melanoma [17,18,19,20]. This result is consistent with the requirement of TRPM8 channels for maintaining proliferation of pancreatic cancer cells and for prevention of replicative senescence [7,9]. A proliferative role of TRPM8 has also been demonstrated in prostate cancer cells [21]. It is interesting to note that menthol-induced activation of TRPM8 leads to reduced cellular viability in a human melanoma cell line [19] and a human urinary bladder cancer cell line [22]. The precise roles of TRPM8 in neoplasia as well as the signaling mechanisms that mediate the cellular functions of TRPM8 remain to be determined. However, the data, thus far, suggest that the aberrantly over-expressed TRPM8 channels may be involved in tumor growth and progression in pancreatic adenocarcinoma.

**Table 2:** Descriptive statistics of pancreatic adenocarcinoma in the tissue microarrays.

Variable	n	%
**Tissues**		
Pancreas	276	98.6%
Liver	1	0.4%
Peritoneum	1	0.4%
Epiploon	1	0.4%
Lymph node (non-regional)	1	0.4%
**Gender**		
Women	110	39%
Men	170	61%
**Histological Grade**		
G1	27	10%
G2	157	57%
G3	89	32%
G4	3	1%
**Primary Tumor (T)**		
T1	9	3%
T2	82	30%
T3	173	64%
T4	6	2%
**Stage**		
I	79	29%
II	175	64%
III	5	2%
IV	15	5%

The expression levels of TRPM8 in pancreatic adenocarcinoma based on the percent coverage by IHC with respect to the intensity and the tumor stages are shown in Table 3.

**Table 3:** The percent coverage ± standard error for expression of TRPM8 in pancreatic adenocarcinoma with respect to the intensity and the tumor stage. The numbers in parentheses represent the patient counts. NA, not applicable.

Tumor stages	Intensity of anti-TRPM8 immunoreactivity				
	0	1	2	3	4
**I**	0 (1)	65.0% ± 4.2% (18)	72.7% ± 2.2% (52)	82.5% ± 3.1 (8)	NA (0)
**II**	NA (0)	59.4% ± 4.0% (43)	79.0% ± 1.8% (85)	86.7% ± 1.7% (46)	90.0% (1)
**III**	NA (0)	NA (0)	60.0% ± 12.2% (4)	90.0% (1)	NA (0)
**IV**	NA (0)	76.7% ± 18.6% (3)	65.0% ± 10.6% (6)	76.7% ± 6.2% (6)	NA (0)

### 3.4. TRPM8 is Required for Invasion in Pancreatic Adenocarcinoma Cells

The process of cell migration is essential for physiological functions, and the ability of cell invasion represents one of the hallmarks of cancer. In our previous report, the pancreatic adenocarcinoma cell lines with RNA interference-mediated repression of *TRPM8* exhibited significantly reduced ability to proliferate and underwent replicative senescence [7,9]. However, the role of TRPM8 channels in pancreatic cancer cells invasion was previously unknown.

**Figure 5:**
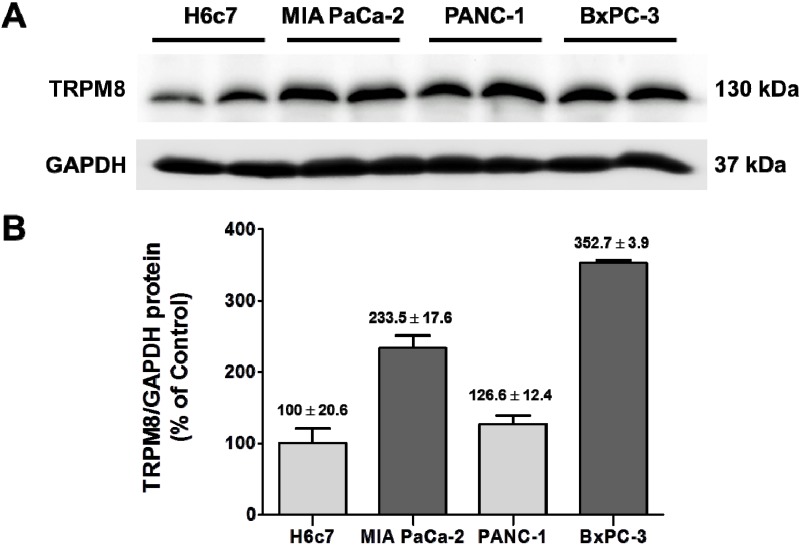
Over-expression of TRPM8 protein in human pancreatic adenocarcinoma cell lines. (**A**) The protein levels of TRPM8 in H6c7, MIA PaCa-2, PANC-1, and BxPC-3 cells were determined using immunoblotting with anti-TRPM8 antibodies. The GAPDH protein levels were analyzed as internal controls. (**B**) The relative protein levels of TRPM8 are expressed as % (mean ± standard error) of that in H6c7.

To gain further understanding of the significance of the aberrantly expressed TRPM8 in pancreatic adenocarcinoma, we determined the ability of cancer cells to invade by inhibiting its expression. The human pancreatic adenocarcinoma cell lines, BxPC-3 and MIA PaCa-2, were employed in this experiment. As demonstrated in our previous report, the relative levels of *TRPM8* mRNA in BxPC-3 and MIA PaCa-2 are increased as compared to that in the non-cancerous pancreatic ductal epithelia H6c7 [7]. In agreement with that finding, TRPM8 protein is expressed at higher levels in those pancreatic cancer cell lines than H6c7 (Figure 5). To down-regulate expression of TRPM8, BxPC-3 and MIA PaCa-2 cells were transfected with shRNA directed against TRPM8. Analysis using semi-quantitative PCR indicated that anti-*TRPM8* shRNA repressed *TRPM8* mRNA levels in these cancer cells by up to 70% as compared to non-targeting control shRNA.

**Figure 6:**
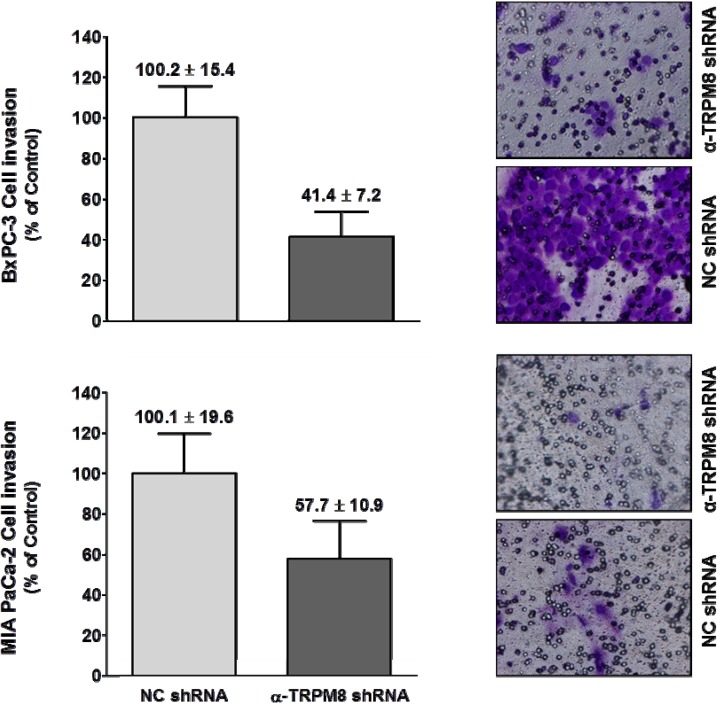
Short hairpin RNA-mediated silencing of *TRPM8* impaired invasion of pancreatic adenocarcinoma cells. BxPC-3 and MIA PaCa-2 cells transfected with either anti-*TRPM8* shRNA or NC shRNA were analyzed for cell invasion using the trans-well assay. Cell invasion is expressed % (mean ± standard error) of that in the cells transfected with control shRNA. Representative images of the invaded cells stained with crystal violet are shown at 200× magnification.

To assay for cell invasion, BxPC-3 and MIA PaCa-2 cells were transfected with either anti-*TRPM8* shRNA to down-regulate TRPM8 expression or non-targeting shRNA as controls. The transfected cells were then seeded in trans-well inserts with filters coated with a solubilized tumor-associated basement membrane matrix (Matrigel™), and FBS was used as a chemoattractant. In parallel with the cell invasion assay, cell viability was determined for the cells transfected with either anti-*TRPM8* shRNA or control shRNA by trypan blue exclusion, and cell viability was almost 100%. In a trans-well assay using 10% FBS as a chemoattractant, RNA interference-mediated silencing of *TRPM8* impaired migration of the BxPC-3 and MIA PaCa-2 cells by about 60% and 45%, respectively (Figure 6).

These results indicate that TRPM8 is required for invasion in pancreatic cancer cells. Consistent with these data, menthol-induced activation of TRPM8 enhances human glioblastoma cell migration in response to hepatocyte growth factor [23]. In our experiments using pancreatic adenocarcinoma cell lines, FBS presumably contains the stimulating factors of cell invasion. This is supported by the fact that pancreatic cancer cells exhibited minimal invasion in the absence of FBS (14%) *vs.* 100% in the presence of 3% FBS. However, the pro-invasion factors present in FBS, as well as the signaling mechanisms that mediate TRPM8 channels-regulated invasion in pancreatic cancer cells, remain to be determined. We hypothesize that the TRPM8 channels in cancer cells perturb Ca^2+^ homeostasis, leading to modulation of cell-adhesion molecules and cell-motility mediators, and consequently enhanced ability of invasion. Taken together, results of this study suggest that the aberrantly expressed TRPM8 channels contribute to the growth and metastasis in pancreatic cancer.

## 4. Conclusions

In this study, we examined the expression of TRPM8 in human pancreatic tissues by immunohistochemistry and investigated its role in pancreatic cancer cells invasion. We presented evidence that expression of TRPM8 protein in normal pancreatic tissues is cell-type specific, primarily in the ductal cells, centroacinar cells, and endocrine cells. TRPM8 is aberrantly expressed in chronic pancreatitis and PanINs, and various types of pancreatic tumors. In the majority of specimens of pancreatic adenocarcinoma, the expression levels of TRPM8 are relatively moderate or high. A positive correlation is demonstrated in pancreatic adenocarcinoma between the expression levels of TRPM8 and tumor size/stages. Moreover, our data suggest that TRPM8 channels are required for pancreatic cancer cells invasion. Taken together with our previously demonstrated role of TRPM8 channels in maintaining proliferation and preventing replicative senescence in pancreatic cancer cells, we propose that the aberrantly expressed TRPM8 plays a contributory role in the growth and metastasis in pancreatic adenocarcinoma. Furthermore, we suggest that TRPM8 channels can be potentially developed as a clinical biomarker and therapeutic target in pancreatic adenocarcinoma.
